# Posterior Fixation Combined with Vertebroplasty or Vertebral Column Resection for the Treatment of Osteoporotic Vertebral Compression Fractures with Intravertebral Cleft Complicated by Neurological Deficits

**DOI:** 10.1155/2019/4126818

**Published:** 2019-12-14

**Authors:** Hongyu Wei, Chunke Dong, Yuting Zhu

**Affiliations:** ^1^Department of Orthopaedic Surgery, China-Japan Friendship Hospital, 2 Yinghuadong Road, Chaoyang District, Beijing 100029, China; ^2^Beijing University of Chinese Medicine, 11 North Third Ring Road East, Chaoyang District, Beijing 100029, China; ^3^Department of Education, China-Japan Friendship Hospital, 2 Yinghuadong Road, Chaoyang District, Beijing 100029, China

## Abstract

**Purpose:**

The aim of the current study was to evaluate the relative benefits of posterior fixation combined with vertebroplasty (PFVP) or vertebral column resection (PVCR) for osteoporotic vertebral compression fractures (OVCFs) with intravertebral cleft (IVC) complicated by neurological deficits.

**Methods:**

From June 2010 to January 2015, 45 consecutive patients suffering OVCFs with IVC and spinal cord injuries were treated with PFVP or PVCR in our department. The visual analogue scale (VAS) score, anterior vertebral height (AVH), posterior vertebral height (PVH), local kyphotic angle (LKA), and neurologic function were evaluated and compared, and the operative duration, blood loss, and complications were also recorded.

**Results:**

They all achieved excellent pain relief, vertebral height recovery, and kyphosis correction one month after surgery, and no significant differences existed between the two groups. No significant differences were observed between the 1-month postoperative and final follow-up VAS, AVH, and LKA values in the PVCR group (*P* > 0.05), while AVH and LKA worsened in the PFVP group at the final follow-up (*P* < 0.05). Similarly, the initial improvements in VAS scores decreased over time (*P* < 0.05). Neurologic function improved in both groups, and no significant differences were observed between the 2 groups either preoperatively or postoperatively (*P* > 0.05). The blood loss and operative duration were significantly lower in the PFVP group than those in the PVCR group (*P* < 0.05).

**Conclusion:**

Compared with PVCR, PFVP had equivalent short-term clinical outcomes with less blood loss and operative duration which can be very beneficial for treating elderly patients with extreme comorbidities in this condition. However, based on the long-term efficacy of pain relief, vertebral height maintenance, and deformity correction, PVCR is a more reasonable choice.

## 1. Introduction

The intravertebral cleft (IVC), which was first described by Maldague et al. [1], has long been considered the result of bone osteonecrosis [2] and does not occur more frequently in osteoporotic vertebral compression fractures (OVCFs) than in the femoral and humeral heads [3]. Patients with IVC often present with a transverse, linear, or semilunar radiolucent shadow, indicating the collection of air inside the vertebral body [2–4]. However, several studies have also observed fluid accumulation within nonhealing intervertebral clefts in patients with benign OVCFs, which depends on the position of the patient secondary to the extension momentum in the supine position [5–7]. Thus, some scholars propose that this alternating air or fluid phenomenon can be considered indicative of a microinstability at the site of the cleft, which ultimately forms a pseudarthrosis, also known as Kummell's disease. Currently, the incidence of IVC was different in OVCFs patients in the literature reports [8–11] and the different results may be caused by the following factors: (1) The sensitivities of plain radiograph, computed tomography (CT), and magnetic resonance imaging (MRI) in detecting IVC are different. Wu et al. [9] found that the accuracy of detecting IVC with plain radiographs, CT scans, and MRI scans was 35.4%, 89.3%, and 83.3%, respectively. Moreover, McKiernan et al. [12] found that the accuracy of plain radiography for patients in a standing position was 16%, while that of patients in a supine position increased to 64%. (2) The incidence of IVC is also dependent on the patients enrolled in the particular study. Kumpan et al. [13] calculated a frequency of 0.85% by reviewing the radiographs of 2,000 patients with vertebral compression fractures. When patients with only OVCFs were selected, an even higher frequency of 10–48% was observed [14, 15]. IVC is probably not pathognomonic of Kummell's disease but may be highly suggestive of it, because IVC is also found in various other spinal conditions, including infections, prolonged use of steroids, multiple myeloma, and malignancies [16–18].

Owing to the progression of kyphosis with vertebral collapse and intravertebral instability at the cleft site, patients with advanced-stage Kummell's disease are more susceptible to neurological deficits [19]. Minimally invasive procedures, such as percutaneous vertebroplasty (PVP) or percutaneous kyphoplasty (PKP), have been widely applied once conservative treatment fails for OVCFs with IVC [10, 20–23]. Although a few studies have confirmed that vertebroplasty could be an alternative intervention for the effective and safe treatment of OVCFs with IVC complicated by neurological deficits, patients with low intravertebral stability and severe spinal cord injuries should be considered candidates for reconstructive surgery [19]. Currently, several open surgery options, mainly consisting of posterior fixation combined with vertebroplasty (PFVP) or vertebral column resection (PVCR) for IVC complicated by neurological deficits, have been reported in the literature [20, 24–26]. However, the preferred surgical option remains controversial. To the best of our knowledge, no previous comparative studies have investigated PFVP and PVCR to treat OVCFs with IVC complicated by neurological deficits. Thus, we compared the efficacies of PFVP and PVCR in treating this condition, and this study may provide a reference for the selection of therapeutic methods.

## 2. Materials and Methods

### 2.1. Patients

From June 2010 to January 2015, 145 consecutive patients with confirmed OVCF with IVC in our department were included; 45 of these patients suffered from spinal cord injuries and underwent an open surgery procedure. Based on the different surgical interventions used for treatment, the patients were divided into two groups (PFVP or PVCR). The demographic data (patient age, sex, lesion segment, bone mineral density (BMD), fusion levels, operative duration, blood loss, and duration of follow-up) of the 2 groups are presented in Table 1. OVCFs with IVC were radiographically diagnosed with the following criteria [4, 19, 21, 27]: an IVC sign showing a transverse, linear, semilunar, or irregular region with radiolucent shadows on CT and/or plain radiographs of the spine; a fluid-containing IVC showing low-signal intensity on T1-weighted images and high-signal intensity on T2-weighted images and/or short-tau-inversion-recovery (STIR) MR images; an air-containing IVC showing homogenous low-signal intensity on T1-weighted, T2-weighted, and/or STIR images; or both air and fluid-filled IVC showing mixed high- and low-signal intensity on T2-weighted and/or STIR images. The inclusion criteria were as follows: (1) osteoporosis identified before the operation by dual-energy X-ray absorptiometry (DXA) and calculated *T*-scores (*T*-score <−2.5 was defined as osteoporosis); (2) single-level neurological deficits in OVCFs complicated by IVC; (3) follow-up period of at least 2 years; and (4) regular radiologic studies including preoperative and postoperative (immediately, 1 years, and 2 years) scans. The exclusion criteria were as follows: (1) multiple-level OVCFs; (2) nonosteoporotic vertebral compression fractures such as pathologic fractures due to multiple myeloma, cancer metastasis, or infection; and (3) multiple severe medical conditions, such as cardiopulmonary anomaly, hepatic failure, or renal failure, which do not let the patient tolerate general anesthesia.

### 2.2. Operative Procedures

#### 2.2.1. PFVP Group

The patients in this group received general anesthesia and were placed in a prone position. After the patients were positioned under the C-arm X-ray, a standard posterior midline approach with subperiosteal stripping was used to expose the spinous processes, lamina, and facet joint. Then, pedicle or cortical bone trajectory [28] screws were inserted bilaterally 1–3 levels above and below the diseased vertebra. Then, the screws were fixed with 2 rods to reduce the vertebral height. Performing decompressive laminectomy of the diseased vertebra was critical, and intraoperative exploration showed no spinal cord compression. A unilateral transpedicular working channel was established by a cannula and trocar system, and polymethylmethacrylate (PMMA) cement was slowly injected into the diseased vertebra through vertebroplasty needles under fluoroscopic monitoring. Posterolateral fusion with autogenous bone grafts from the decompression laminectomy was performed. A drainage tube was left in the surgical field, and the wound was closed in layers.

#### 2.2.2. PVCR Group

In this group, the patients were also placed in a prone position on a frame after general anesthesia. A standard midline incision was made, and subperiosteal dissection was performed to expose the diseased vertebra and the adjacent levels, including the lamina, transverse processes, facet joints, and the costotransverse joints of the thoracic spine. Pedicle screws were inserted 1-2 levels above and below the diseased vertebra. A laminectomy was then performed to decompress and completely exposed the dural sac. A temporary stabilizing rod was fixed on one side of the pedicle screws. On the contralateral side, the facet joints and rib transverse joints of the diseased vertebra were removed to reveal the pedicle. Then, the pedicle and fractured body, including the superior and inferior disks, were removed by rongeurs, osteotomes, curettes, or a high-speed drill, and a bone specimen was retrieved from the intravertebral cleft of the diseased vertebra and sent for pathological examination. A titanium mesh cage packed with autologous bone chips was inserted into the corpectomy site. After reducing the kyphotic changes, two rods were tightened in an alternating manner. Posterolateral fusion with autogenous bone grafts from the resected bone was performed on the secured vertebral laminae, facets, and transverse laminae. A drainage tube was left in the surgical field, and the wound was closed in layers.

Somatosensory-evoked potentials (SEPs) and motor-evoked potentials (MEPs) were monitored in the spinal cord throughout the entire surgical procedure. The patients were allowed to walk 1 week after surgery while wearing a brace. Orthosis was used for at least 3 months until complete bone fusion was achieved.

### 2.3. Radiological and Clinical Evaluation

**Figure 1:**
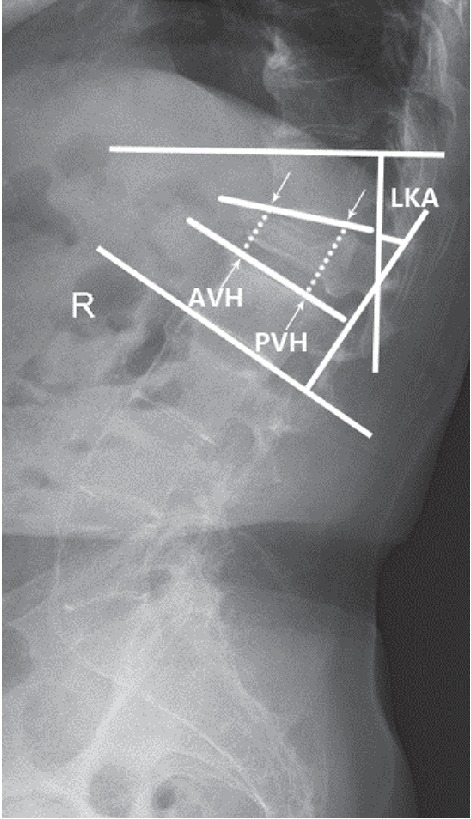
The degree of local kyphotic angle (LKA) was measured from the upper endplate of the instrumented vertebra to the lower endplate of the instrumented vertebra below the fractured level. The anterior vertebral height (AVH) and posterior vertebral height (PVH) were measured as the distance between the upper and lower endplates at the anterior wall and posterior wall of the vertebral body, respectively.

Anteroposterior and lateral radiography, CT, and MRI data were collected preoperatively, one week after surgery, and at the final follow-up. The local kyphotic angle (LKA), which was determined by Cobb's method, was measured as the angle between the superior endplate of the adjacent upper vertebra and the inferior endplate of the lower vertebra. The vertebral height was measured as the height of the anterior and posterior margins of the diseased vertebral body (anterior vertebral height (AVH) and posterior vertebral height (PVH)) as described by Lee et al. [26]. The AVH, PVH, and LKA were assessed before and after surgery (Figure 1). To correct the magnification ratio on radiographs acquired preoperatively and postoperatively, we used the Picture Archiving and Communication Systems (PACS) (Carestream Health, Inc., Shanghai, China) to measure the imaging data in our hospital; the data were obtained by taking the average of two measurements obtained by two independent senior spine surgeons.

A visual analogue scale (VAS), which ranged from 0 (no pain) to 10 (maximal pain), was used to assess pain severity, especially when the patient changed positions. The measures were evaluated preoperatively, 1-month postoperatively, 1 year after the surgery, and 2 years after the surgery (the final follow-up). The American Spine Injury Association (ASIA) grading system was used to assess the neurological status preoperatively and at the final follow-up. The operative duration, volume of blood loss, complications such as intraoperative injuries to the spinal cord and dura, postoperative complications including wound infection, the development of nonunion, and kyphotic aggravation and instrument failure were also recorded.

### 2.4. Statistical Analysis

All analyses were performed using SPSS20.0 software (SPSS Inc., Chicago, USA). All data were expressed as the mean ± standard deviation (SD) for parametric analyses. The baseline characteristics of the two groups were compared using paired *t*-tests or Fisher's exact tests. The between-group difference in AVH, PVH, LAK, and VAS from preoperative follow-up to final follow-up was evaluated by MANOVA when the data conform to a normal distribution. The within-group change was assessed by repeated measurement analysis. Wilcoxon test was used for nonconformable normal distribution data. A value of *P* < 0.05 was considered to indicate a statistically significant difference.

## 3. Results

### 3.1. Patient Characteristics

**Figure 2:**
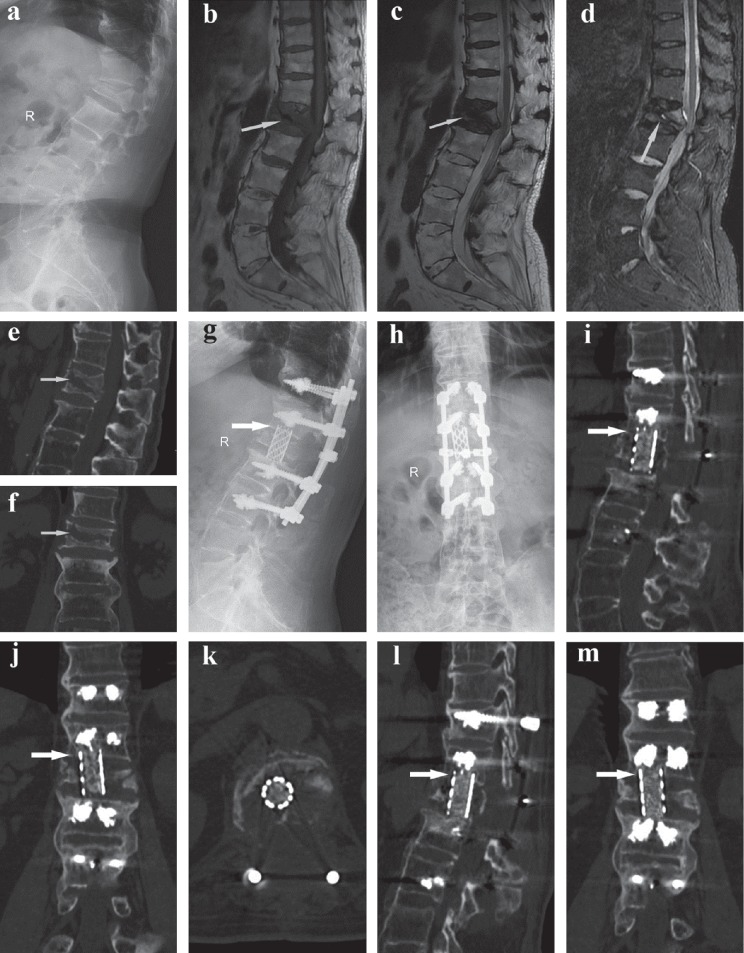
A 67-year-old male patient experienced OVCFs with IVC complicated by neurological deficits and underwent PVCR treatment. (a) Preoperative sagittal plain radiographs revealed collapsed fracture at L1 and kyphosis. (b, d) Sagittal T1- and T2-weighted MRI and STIR MRI scans showed a decreased signal in the intravertebral cleft in L1. (e, f) Coronal and sagittal CT showed an IVC in L1. (g, h) Immediate postoperative plain radiographs showed corpectomy, screw fusion, increased anterior vertebral height, and restored kyphosis. (i–m) Coronal and sagittal CT showed no reduction in vertebral height and no kyphosis recurrence at the 1- and 2-year follow-up examinations.

**Figure 3:**
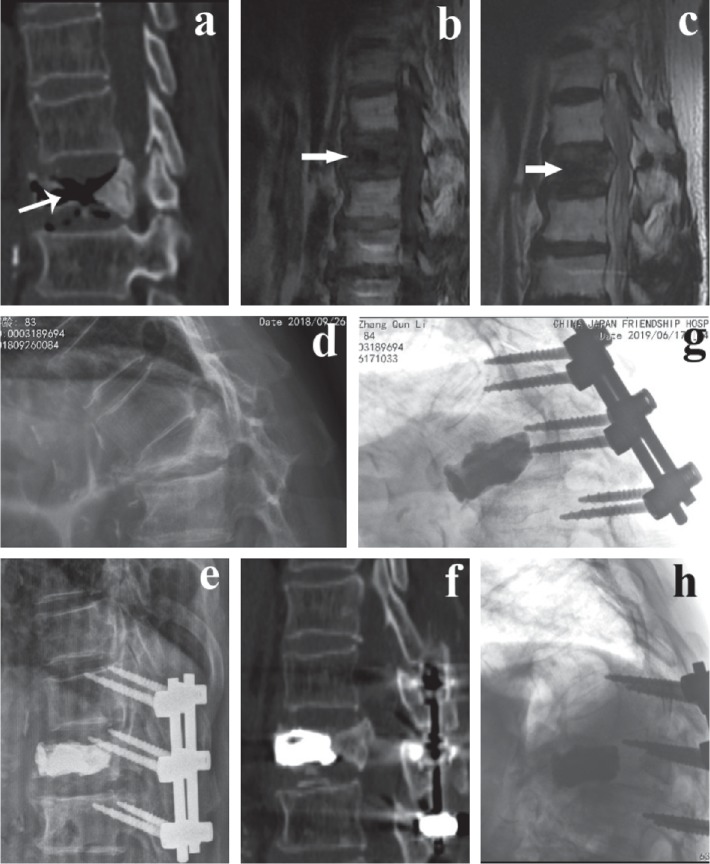
An 83-year-old male patient underwent PFVP due to L1 OVCFs with IVC complicated by neurological deficits. (a–d) Preoperative sagittal plain radiographs, T1- and T2-weighted MRI, and CT revealed an IVC and spinal cord compression at the L1 level. (e, f) Immediate postoperative plain radiographs and CT showed bone cement in the vertebral body with increased anterior vertebral height and resolved kyphosis. (g, h) Plain radiographs showed that displacement of the bone cement developed 1.5 years after surgery.

**Figure 4:**
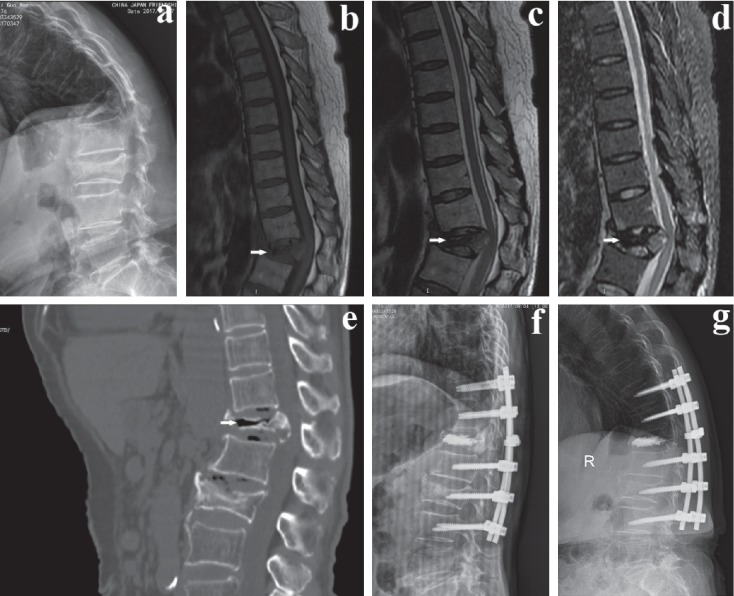
A 76-year-old male patient underwent PFVP because of L1 OVCFs with IVC complicated by neurological deficits. (a–e) Preoperative sagittal plain radiographs, T1- and T2-weighted MRI, STIR MRI, and CT showed an IVC and spinal cord compression at the L1 level with a collapsed fracture and kyphosis. (f) Immediate postoperative plain radiographs showed bone cement in the vertebral body with increased anterior vertebral height and resolved kyphosis. (g) Lateral radiographs showed kyphosis recurrence after 2 years.

A total of 45 patients were enrolled in our study, including 21 who were in the PFVP group and 24 who were in the PVCR group. None of the baseline parameters including patient age, sex, lesion segment, BMD, fusion levels, duration of follow-up, and preoperative LKA, AVH, PVH, and VAS scores were significantly different between the two groups (*P* > 0.05) (Table 1). Some illustrative cases are shown in Figures 2–4.

### 3.2. Radiological Findings

All radiological parameters, including LKA, AVH, and PVH, were determined preoperatively, postoperatively, 1 month after surgery, and at the final follow-up. The preoperative LKA, AVH, and PVH values were not significantly different between the PFVP and PVCR groups (30.45 ± 12.18° vs. 30.12 ± 10.46°, 17.26 ± 5.82 mm vs. 19.00 ± 5.50 mm, and 29.17 ± 4.11 mm vs. 29.79 ± 4.90 mm, respectively; *P* > 0.05). The LKA, AVH, and PVH values significantly improved after surgery (*P* < 0.05), and no significant differences existed between the two groups one month after surgery (10.17 ± 8.63° vs. 10.48 ± 7.47°, 29.81 ± 6.99 mm vs. 30.16 ± 6.13 mm, and 33.01 ± 5.40 mm vs. 32.87 ± 4.46 mm, respectively; *P* > 0.05). In the PVCR group, the 1-month postoperative and last follow-up LKA and AVH values were not significantly different (10.48 ± 7.47° vs. 11.65 ± 7.51° and 30.16 ± 6.13 mm vs. 29.73 ± 7.41 mm, respectively; *P* > 0.05). However, in the PFVP group, compared to the 1-month preoperative levels, an average 13.3° loss in LKA correction and 10.2 mm AVH collapse were found at the final follow-up (23.51 ± 9.3° vs. 10.17 ± 8.63° and 19.63 ± 4.11 mm vs. 29.81 ± 6.99 mm, respectively; *P* < 0.05). No significant losses of PVH occurred between the 1-month preoperative and final follow-up in the two groups, and the PVH values of the two groups were not significantly different (*P* > 0.05).

### 3.3. Surgical and Clinical Results

**Figure 5:**
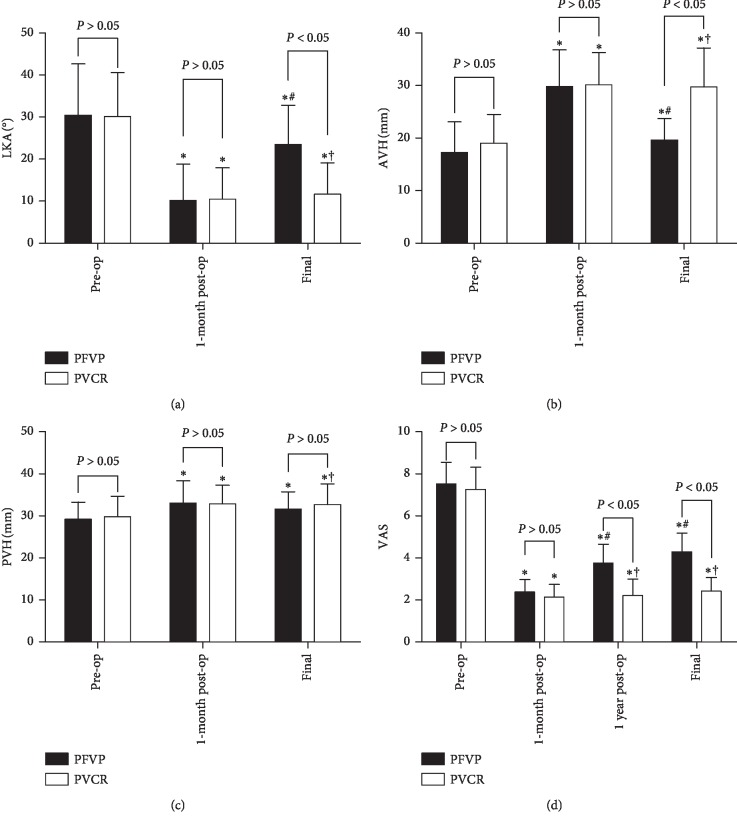
Radiological outcomes and VAS scores. (a–d) ^*∗*^*P* < 0.05 compared with the preoperative data. ^#^*P* < 0.05 compared with the 1-month postoperative data. ^†^*P* > 0.05 compared with the 1-month postoperative data.

The follow-up period ranged from 19 to 47 months (mean, 31.29 ± 7.98 months) in the PFVP group and from 17 to 49 months (mean, 32.68 ± 8.72 months) in the PVCR group. The operative duration in the PFVP group (150.48 ± 23.58 minutes) was significantly lower than that in the PVCR group (223.08 ± 28.78 minutes; *P* < 0.05). The volume of blood loss in the PFVP group (252.62 ± 37.94 mL) was significantly lower than that in the PVCR group (413.25 ± 84.50; *P* < 0.05) (Table 1). The mean VAS score decreased significantly after surgery in both groups. The mean VAS scores in the PFVP group and PVCR group preoperatively were 7.52 ± 1.03 and 7.25 ± 1.07, and 1 month postoperatively, the scores dropped to 2.38 ± 0.59 and 2.13 ± 0.61, respectively (*P* < 0.05, Figure 5). The postoperative follow-up showed that the mean VAS scores 1 year and 2 years after surgery were still lower than the preoperative scores of both groups. The long-term follow-up VAS score did not significantly change in the PVCR group, but the score for the PFVP group significantly increased after 2 years (*P* < 0.05, Figure 5). The 1-year and 2-year postoperative VAS scores were significantly different between the 2 groups (*P* < 0.05, Figure 5). Preoperatively, no significant difference in neurological status existed between the two groups (*P* < 0.05). In addition, each patient's neurological function improved by at least one or two levels, and no significant differences were found during the final follow-up between the two groups (*P* < 0.05) (Table 2).

### 3.4. Complications

No serious intraoperative or postoperative complications, including neurologic injury or instrumentation failure, occurred in the two groups. In the PFVP group, 2 patients had asymptomatic cement leakage. One patient had a urinary tract infection that was cured by conservative treatment. One patient developed displacement of the bone cement during the follow-up, and we used conservative treatment because no neurologic deficits were present. In the PVCR group, 3 patients had dural tears with cerebrospinal fluid leakage, which were covered intraoperatively by fascia tissue, and a lumbar drainage tube was placed and removed after 7 days. One superficial surgical site infection was treated by antibiotics, and one deep wound infection resolved after debridement. In this study, no significant difference was observed regarding the complications between the 2 groups (*P* > 0.05; Table 1).

## 4. Discussion

In recent years, PKP and PVP seem to be the standard for treating OVCFs with IVC because these techniques have advantages of satisfactory pain relief and vertebral height recovery via a relatively minimally invasive approach. However, several studies [8, 10] have reported a high incidence of recollapse of the augmented vertebrae during long-term follow-up after PKP to treat OVCFs with IVC, and some authors [27, 29] have proposed that the preoperative IVC might be an independent risk factor for recollapse of the augmented vertebrae after PKP or PVP. Although Nakamae et al. [19] proposed that PVP could be an alternative intervention for the effective and safe treatment of OVCFs with IVC associated with neurological deficits, more attention should be paid to performing PKP or PVP for OVCFs with IVC and the development of dynamic instability.

Therefore, open surgery for spinal cord decompression and spine stabilization is more reasonable for these patients. The surgical options include anterior, posterior, and combined anterior and posterior procedures; however, the preferred surgical procedure is under debate [30]. In recent years, a posterior approach has been recommended because posterior fixation provides relatively stable fixation to prevent implant-related complications. Patients with OVCFs with IVC are always of an advanced age, have serious comorbidities, and do not easily tolerate multiple surgical methods, and the development of a single-stage treatment method is required. Cho [25] retrospectively analyzed 22 patients who underwent PVCR and mesh cage insertion and believed that this surgical approach was considered an effective option for advanced Kummell's disease with neurological deficits. The author believed that this procedure provided anterior support to minimize posterior pedicle screw stress and to increase the fusion rate. However, this method requires relatively high-level surgical skills and a long learning curve [25]. Thus, for this condition, some scholars have recently recommended PFVP, a relatively simple and minimally invasive approach that does not need to address the anterior lesion [20, 26]. Lee et al. [26] showed that PFVP is an effective treatment for OVCFs with IVC complicated by neurologic symptoms; however, the authors observed an average 4.5° loss of correction in the kyphotic angle at the final follow-up. Several studies have also reported that dislodgement of the injected PMMA cement occurred with concomitant recollapse of the IVC vertebrae, possibly because vertebroplasty does not offer sufficient support to the compressed vertebra [31, 32]. Therefore, whether PFVP or PVCR is more appropriate for OVCFs with IVC complicated by neurological deficits is under debate. To the best of our knowledge, no current literature reports compare these two surgical methods.

Although both groups achieved similar short-term clinical outcomes, including pain relief, vertebral height recovery, and malformation improvement (*P* > 0.05), the long-term effects were different; the PFVP group had significant losses of AVH and LKA progression during the 2-year follow-up compared to the PVCR group (*P* < 0.05), which is different from previous studies [20, 33–35]. As kyphosis was aggravated over time, patients in the PFVP group complained of more severe back pain than those in the PVCR group (*P* < 0.05), which is consistent with the VAS results observed in our study. This result may be attributed to recurrent instability at the augmented vertebral body. In our series, the PVCR approach included a mesh cage insertion and augmented segmental fixation, thus providing fusion 1-2 levels above and below the diseased vertebra, reducing the number of fusion segments, and influencing the long segmental spinal function. Simultaneously, since the augmented segmental fixation provided firm integration and anterior support to reduce the pedicle screw stress that arises from osteoporosis, no loss of AVH and LKA progression were observed in the PVCR group (*P* > 0.05). In contrast, the PFVP approach failed to provide effective anterior support because achieving a mechanical interlock between the normal trabecular bone and PMMA with this method is difficult during vertebroplasty, which could be explained by the following reasons: First, the exothermal and toxic effects of PMMA may interfere with the healing process of osteonecrosis and fractures rather than providing stabilization. PMMA is not a bioactive filler material and, as such, cannot be replaced by new bone formation [36]. Second, due to the presence of IVC, a solid collection of cement often forms during PVP or PKP. The lack of distribution might result in less contact between the cement and cancellous bone, and the cement may concentrate stress on the surrounding fragile bones, which could promote recollapse around the cement and trigger LKA rebound after surgery [10]. In our study, since posterolateral fusion fixation with autogenous bone grafts was performed in both groups, no loss of PVH was observed at the final follow-up in either group, which indicated that the progressive kyphosis developed due to the lack of support from the anterior spine.

In our retrospective study, the 1-month postoperative VAS score, LKA, and vertebral height (AVH and PVH) improved significantly compared to the preoperative values in both the PFVP and PVCR groups (*P* < 0.05), which is in accordance with the findings of previous studies [20, 25, 26, 35]. Furthermore, the neurological function of each patient improved by at least one or two levels at the final follow-up, and no significant differences existed regarding the preoperative and postoperative neurological statuses between the two groups. These results show that PFVP and PVCR are both effective treatments for OVCFs with IVC complicated by neurological deficits and do not pose risks to the chest or abdominal organs and thus would not cause serious pulmonary or gastrointestinal complications in elderly patients. In addition, most surgeons are familiar with the posterior approach. Regardless of the long-term efficacy of these two methods for pain relief or the kyphosis correction, the PVCR group had a longer operative duration and higher volume of blood loss than the PFVP group (*P* < 0.05), which can be attributed to the lack of anterior osteotomy and debridement in the PFVP group. No significant differences were observed regarding the complications between the 2 groups in our study. Based on the above points, PFVP seems to be more suitable for OVCF with IVC complicated by neurological deficits than PVCR, especially for elderly patients with comorbidities. However, the long-term efficacy of the two methods differed, and the PFVP group had a significant loss of AVH, progression of LKA, and recurrence of back pain compared to the PVCR group (*P* < 0.05). Therefore, we recommend PFVP for the treatment of OVCFs with IVC complicated by neurological deficits to improve the neurological symptoms in patients with severe comorbidities. Otherwise, PVCR is a more reasonable approach in this situation with better long-term results than PFVP.

Some limitations of this study need to be addressed. First, we did not establish a control group that underwent conservative treatment. Second, recruitment bias seemed to be inevitable; the site of the lesion and the fusion levels were considered when selecting patients, which could potentially change the outcome of this study. Third, our research was retrospective, and the sample size was small. A prospective, randomized controlled study could better assess the clinical outcomes of PFVP and PVCR for the treatment of OVCFs with IVC complicated by neurological deficits.

## 5. Conclusions

Compared with PVCR, PFVP had equivalent short-term clinical outcomes and radiographic findings with decreased blood loss and a shorter operative duration, which can be very beneficial for elderly patients with comorbidities, to treat OVCFs with IVC complicated by neurological deficits. Based on the long-term efficacy of PVCR in relieving back pain, maintaining vertebral height, and correcting kyphosis, PVCR is a more reasonable choice than PFVP.

## Figures and Tables

**Table 1:** Comparison of baseline characteristics in the PFVP and PVCR groups.

Patient characteristics	PFVP (*n*=21)	PVCR (*n*=24)	*P* values
Age (year)	73.76 ± 7.65	72.33 ± 7.25	0.524
Gender (M/F, *n*)	5/16	6/18	0.926
BMD (*T*-score)	−3.20 ± 0.50	−3.32 ± 0.48	0.484
Lesion segment (*n*)	T11 (6), T12 (8), L1 (7)	T10 (1), T11 (8), T12 (10), L1 (5)	0.798
Fusion levels	4.95 ± 1.16	5.08 ± 1.14	0.705
Follow-up (months)	31.29 ± 7.98	32.68 ± 8.72	0.587
Preoperative LKA (°)	30.45 ± 12.18	30.12 ± 10.46	0.922
Preoperative AVH (mm)	17.26 ± 5.82	19.00 ± 5.50	0.308
Preoperative PVH (mm)	29.17 ± 4.11	29.79 ± 4.90	0.653
Preoperative VAS	7.52 ± 1.03	7.25 ± 1.07	0.402
Preoperative ASIA	B (1), C (8), D (12)	B (2), C (10), D (12)	0.836
Operative duration (min)^*∗*^	150.48 ± 23.58	223.08 ± 28.78	<0.001
Blood loss (mL)^*∗*^	252.62 ± 37.94	413.25 ± 84.50	<0.001
Complications	4	5	0.881

^*∗*^ *P* < 0.05 for the comparison between the two groups.

**Table 2:** Comparison of preoperative and postoperative neurological statuses in the two groups.

Group	Preoperative ASIA					Postoperative ASIA				
	A	B	C	D	E	A	B	C	D	E
PFVP (*n*=21)		1	8	12			0	2	4	15^*∗*^
PVCR (*n*=24)		2	10	12			1	3	6	14^*∗*^
*χ* ^2^	0.357					1.472				
*P*	0.836					0.858				

^*∗*^ *P* < 0.05 for the comparison between preoperative and postoperative statuses.

## Data Availability

The data used to support the findings of this study are available from the corresponding author upon request.
